# Effect of early clinical exposure to GRIT among Indian undergraduate dental students

**DOI:** 10.6026/9732063002001233

**Published:** 2024-10-31

**Authors:** Noopur Kokane, Abhay Datarkar, Sachin Khatri, Jyoti Manchanda, Shilpa Warhekar, Aniket Dhote

**Affiliations:** 1 Department of Public Health Dentistry Government Dental College and Hospital, Nagpur, India; 2 Department of Oral Maxillofacial surgery Government Dental College and Hospital Nagpur, India; 3 Department of Orthodontics Government Dental College and Hospital, Nagpur, India

**Keywords:** Dental education, early clinical exposure, grit, perseverance, passion, student motivation

## Abstract

Dental education is acknowledged for its inherent stress, demanding a multifaceted skill set from students. Early Clinical Exposure (ECE) has emerged as a potential tool to enhance students' interest and perseverance in the face of challenges. Therefore, it is of interest to explore the influence of ECE on GRIT levels among first-year dental students. GRIT is the combination of passion and perseverance for long-term goals. It reflects an individual's ability to stay committed to their objectives despite challenges or setbacks. GRIT is considered a key predictor of success, especially in demanding fields like education, where sustained effort is crucial. A modified 12-item GRIT survey was electronically administered to first-year dental students, measuring their perseverance and passion for long-term goals. The study also investigated potential confounding factors such as age, gender and whether dentistry was the first choice for undergraduate studies. The GRIT scale's internal consistency was found to be high (Cronbach's α = 0.88) and test-retest reliability was established (Intra class Correlation Coefficient = 0.98). Data shows a statistically significant positive correlation between early clinical exposure and GRIT levels in first-year dental students. The majority of respondents demonstrated a substantial degree of grit, with 47.5% categorized as "Very Much Gritty." Interestingly, no individuals were classified as "Not Gritty at all." Additional analyses explored the influence of age, gender and dentistry as the first choice on GRIT levels. While no significant impact was observed based on these factors; female students exhibited slightly higher GRIT levels than their male counterparts. This study's findings contribute valuable insights to dental education, emphasizing the potential benefits of integrating ECE into curricula. Dental schools and educators are encouraged to recognize the motivational impact of early clinical exposure, fostering GRIT development among students.

## Background:

According to a report by the Global Congress in Dental Education (2008), "Dental Education is considered as a stressful, complex, demanding pedagogical exposure [1]. It involves the acquisition of required academic, clinical and interpersonal skills during learning." Practising dentistry requires clinical and patient management skills, which also add to the stress the students perceive. There are multiple sources of stress for health professions students in academics *e.g.* exams and classroom performance, fear of failure, clinical e.g., productivity demands, complex cases quota fulfilment and personal *e.g.*, work-life balance, family pressure, financial concerns, concern about future dental [2] curriculum has always been difficult and confusing, especially for 1st-year dental students. Primary objective of Dental education is to prepare students for a lifetime of patient care. Competency based learning is introduces so as to that students do not lose their perspective through the years of study.

Dental academicians are constantly looking for ways to improve their interest and perseverance, creating zeal in them to tirelessly towards challenges, maintaining effort and interest over the years despite failure and hardships in progress [3]. One such method is early clinical exposure (ECE) which can be introduced for first-year and Second-year dental students. Early Clinical Exposure (ECE) is used in medical and dental education to introduce students to real-world clinical experiences early in their training. It aims to provide students with opportunities to observe and interact with patients, healthcare professionals and healthcare settings at an earlier stage of their education. ECE provides a clinical context and relevance to basic sciences learning [4]. ECE also facilitates students to get an early involvement in the healthcare environment which acts as motivation and reference point for students, leading to their professional growth [5].

The influence of ECE is difficult to measure as in the academic performance of the first year. Whether early exposure to clinics is beneficial or distracts the students from their regular academic work is hard to assess. No proper scale is available to assess the influence of ECE in the long run and its effects on the holistic development of the students. However, does ECE increase the interest and perseverance of First-year dental students can be measured, by measuring the GRIT of the students. Grit is a psychological trait that refers to an individual's ability to persevere and maintain long-term passion and effort toward achieving their goals. It is characterized by resilience, determination, and a strong work ethic [6]. The concept of GRIT was popularized by psychologist Angela Duckworth, who defined it as the "perseverance and passion for long-term goals [6]. Grit is one of the most important non-cognitive skills that students can acquire to achieve long-term goals, and oppose challenges and barriers they may face during education. GRIT is the perfect scale to measure whether ECE will help first-year dental students in the long term or not. Therefore, it is of interest to report the effect of early clinical exposure to GRIT among Indian undergraduate dental students.

## Methodology:

## Study context:

The study was conducted at Government Dental College and Hospital, Nagpur. Early clinical exposure was recently introduced as clinical postings in the curriculum of first- and second-year dental students. The study involves first-year dental students. The approval was obtained from the institutional ethical committee.

## Study procedure and participants:

Participants were recruited from the pool of first-year dental students at Government Dental College and Hospital, Nagpur. The survey was administered electronically, and informed consent was obtained from all participants prior to their participation. Participants were assured of confidentiality and anonymity throughout the study process. To mitigate the influence of potential confounding variables, demographic information such as age, gender and whether dentistry was the first choice for undergraduate studies was collected from participants. Statistical analyses, including chi-square tests, were performed to assess the association between these variables and GRIT levels. A significance level of p ≤ 0.05 was used to determine statistical significance. Measures to minimize biases in data collection and analysis included clear instructions provided to participants regarding survey completion, reminders sent to non-responders and anonymization of data during analysis. Additionally, efforts were made to ensure the survey questions were unbiased and comprehensible to all participants. Early Clinical Exposure (ECE) is a pivotal component recently integrated into the dental curriculum at Government Dental College and Hospital Nagpur, reflecting a progressive departure from conventional pedagogical norms. Within this innovative framework, first and second-year dental students are systematically allocated to clinical postings in cohorts of 5-6 students. These postings, occurring on the 1st, 3rd and 5th Saturdays, offer students immersive encounters with patients, clinical environments and community engagements tailored to the exigencies of each department.

## Early clinical exposure (ECE):

The ECE curriculum, meticulously structured into four modules over the first two years, endeavors to imbue students with a profound appreciation for the symbiotic relationship between foundational sciences and clinical practice. Central to this ethos are the overarching objectives of ECE, which include fostering students' recognition of the inherent relevance of basic sciences in diagnostic prowess and patient management, cultivating attitudes of professionalism, ethics, and empathy pivotal to the doctor-patient rapport, and engendering an understanding of the socio-cultural dimensions underpinning disease manifestation through the lens of humanities.

## Integral to the design of ECE modules is the recognition of three fundamental elements:

Provision of Clinical Correlation to Basic Sciences Learning: Modules are meticulously crafted to elucidate the intricate interplay between theoretical concepts and their real-world applications, thereby augmenting students' comprehension and retention of foundational knowledge.

## Human contact:

ECE fosters authentic interactions within clinical or social contexts, thereby facilitating experiential learning and nurturing students' clinical acumen and interpersonal skills from the outset. Introduction to Humanities in Medicine: Acknowledging the holistic nature of healthcare delivery, ECE modules afford students opportunities to explore the humanistic dimensions of medicine, enriching their understanding of patient care and fostering empathy and cultural competence.

## Within this dynamic pedagogical milieu, students assume varying roles reflective of their evolving clinical competencies:

## Passive Observer:

Initially thrust into unfamiliar clinical settings, students adopt a passive observational stance, acclimating themselves to departmental dynamics and workflows.

## Active observer:

As students gain familiarity with clinical contexts, they transition into active observers, engaging with clinical scenarios and augmenting their understanding through participatory observation.

## Active performer:

With increasing proficiency, students assume an active role in departmental activities, contributing meaningfully to patient care and procedural tasks under supervision. ECE modules not only afford students a tangible glimpse into the challenges and rewards of clinical dentistry but also serve as catalysts for professional growth and identity formation. By fostering early engagement and empowerment within clinical settings, ECE instils a sense of purpose and direction, emboldening students to traverse the arduous terrain of dental education with resilience, passion and purpose.

## Validations of GRIT scale to measure ECE:

The original Grit questionnaire was modifies to measure the influence of ECE on the GRIT of the students.

[1] We adapted a Focused Group discussion to evaluate the Face and content validity of the questionnaire. Group discussion was conducted between the investigator, dental academicians and Students

[2] A pilot study was conducted on second-year students who had ECE.

[3] To test the reliability and consistency of the questionnaire, internal consistency and test-retest reliability methods were employed. Internal consistency was expressed as a Cronbach's α value and test-retest reliability as an intra-class correlation coefficient (ICC).

[4] The internal consistency of the questionnaire Cronbach alpha 0.88

[5] For test-retest reliability, the same questionnaires were administered on a convenience sample of 42 students on two occasions with a gap of one week. This yields two scores for each participant and the intra-class correlation coefficient is calculated (Table 1).

## Statistical analysis:

Statistical Package for Social Sciences (SPSS) version 20 was used for data analysis. Means were used for continuous variables and percentages for categorical variables. The chi-square test was applied for the association between age, gender and dentistry as first choice and Grit, P value less than or equal to 0.05 was considered significant. (Figure 1 see PDF, Figure 2 see PDF) represents the demographic distribution of the subjects, such as gender and age.

## Results:

Table 2 illustrates the distribution of GRIT levels within the surveyed population. Grittiness is categorized into five levels. The majority of respondents, 47.5%, are categorized as "Very Much Gritty", indicating a strong level of grit. Additionally, 39.0% of respondents fall into the "Gritty" category. This suggests that a significant portion of the population exhibits a substantial degree of grit. The findings also highlight a relatively small percentage of individuals (6.8%) falling into the "Somewhat Gritty" category, while the "Extremely Gritty" category includes 6.8% of the population. Remarkably, there are no individuals classified as "Not Gritty at all." Table 3 presents the distribution of the level of grittiness among different age groups. Table 4 presents the distribution of the level of grittiness among genders, female students are somewhat more gritter than male students but not statistically significant. There is no significant influence of gender or dentistry as the first choice on the grittiness of the students.

## Discussion:

Dental education is an arduous journey that demands not only a strong academic foundation but also resilience, and persistence. Grit, a psychological construct characterized by passion and perseverance toward long-term goals, is essential for success in dental education and practice [7]. Grit is crucial for dental students, who must navigate a demanding curriculum and develop clinical skills. Research has shown a positive relationship between Grit and academic performance [8, 9]. Grit has also been proven to show a positive relationship with resilience [10, 11]. Early clinical exposure has attracted attention in dental education as a means to better prepare students for the practical challenges they will face in their professional careers. ECE helps to bridge the gap between theoretical knowledge and practical application. The results of this study indicate a statistically significant positive correlation between early clinical exposure and GRIT levels in first-year dental students [12]. These findings are consistent with previous research highlighting the advantages of early clinical exposure. Studies have found that early clinical exposure significantly increased students' self-efficacy and their ability to persevere through challenging situations. It also helps in the development of interpersonal skills [13]. The exposure allowed students to connect their academic knowledge to clinical practice, which enhanced their motivation and sense of purpose in their studies [14]. This aligns with our study's findings, suggesting that early clinical exposure can be a motivational factor in increasing students' grit.

Studies have also shown that for the majority of students, dentistry is not the first choice for undergraduate courses, for the majority it's an alternative to medical. For such students' dental curriculums become very stressful, especially for the first two years when they have no contact with the patients [15]. They are more likely to lose their interest and sense of purpose. Early clinical exposure offers students an opportunity to witness the real-world relevance of their education in the first year, which can boost their motivation and commitment. This aligns with the findings of Duckworth and Quinn, who argued that a sense of purpose and passion for long-term goals are central components of grit. Students who can directly observe the impact of their education on patient care may develop a clearer sense of purpose, fostering their GRIT development. Furthermore, the development of practical skills and problem-solving abilities early in their education is crucial for dental students. As highlighted by Sheehy *et al.* these practical skills are closely related to grit, as they require perseverance and resilience to master [15]. The ability to handle complex clinical cases and adapt to changing circumstances can contribute to GRIT development, which is consistence with our study findings. Studies have consistently demonstrated that Early Clinical Exposure (ECE) is a transformative approach in dental education, offering benefits that extend beyond academic learning. By integrating students into clinical settings early in their undergraduate journey, ECE fosters a deeper understanding of theoretical concepts through real-life application, enhancing the overall learning experience [13]. The grit and perseverance required during these formative experiences equip dental students with resilience and empathy, essential traits for thriving in a demanding healthcare environment. Thus, ECE not only enhances academic outcomes but improves professional wellbeing and attitude of dental students [17].

## Limitations of the study:

The study was conducted in a single institute and on a single-year student, thus limiting generalizability. It is a self-report questionnaire, so social desirability bias is the possibility, where respondents who answer the question to show their thinking positive.

## Conclusion:

Early clinical exposure (ECE) can enhance student motivation and GRIT in dental education. Incorporating ECE into curricula may help students persist through academic and clinical challenges. These findings offer valuable insights, encouraging educators to integrate ECE to better prepare students for dental practice.

## Figures and Tables

**Table 1:** Intra-class Correlation Coefficients (ICC) and confidence intervals for grit scale items in early clinical exposure study

**Grit Scale**	**Intraclass Correlation Coefficient**	**95% Confidence Interval**	
		**Lower Bound**	**Upper Bound**
1. Early clinical exposure can help me conquer important challenges I might face in future clinics	0.96	0.928	0.986
2. Exposure to clinic practice sometimes distracts me from my regular studies	0.97	0.953	0.991
3. Due to early clinical exposure my interest in different subjects' changes from time to time	0.91	0.802	0.961
4. Set back faced during early clinical practice postings does not discourage me.	0.95	0.891	0.978
5. I get obsessed with a subject during my clinical posting but later loose interest in it	0.84	0.643	0.931
6. Because of the exposure to clinical practice, I have started to work more harder	0.9	0.792	0.96
7. Early clinical practice postings distract me from my present set goals	0.99	0.977	0.995
8. Because of the early clinical postings, I find it difficult to focus on my present projects / first year curriculum that take more than few months to complete	0.96	0.922	0.984
9. Early clinical posting helps me to finish whatever task I begin concerning my current academic curriculum with ease.	0.96	0.928	0.986
10. Early clinical posting will help me to achieve my long-term goals easily	0.95	0.891	0.978
11. I become interested in new pursuits of early clinical posting more than my first-year curriculum	0.96	0.928	0.986
12. Early clinical postings help me to be more diligent	0.91	0.802	0.961
Total Grit Scale	0.98	0.968	0.994

**Table 2:** Grit score

**S. no**	**Grit score**	**n**	**%**
1	Not Gritty at All (0-1.99)	0	0
2	Somewhat Gritty (2-2.99)	4	6.80%
3	Gritty (3-3.99)	35	39.00%
4	Very Much Gritty (4-4.99)	17	47.50%
5	Extremely Gritty (5)	4	6.80%

**Table 3:** Grit score

**Age**			**Level of grittiness**			**P-value**
	**Not at all Gritty**	**Somewhat Gritty**	**Gritty**	**Very much Gritty**	**Extremely Gritty**	
18yrs	0.00%	0.00%	50.00%	50.00%	0.00%	
19yrs	0.00%	7.10%	35.70%	57.10%	0.00%	0.812
20yrs	0.00%	8.30%	45.80%	33.30%	12.50%	
21yrs	0.00%	0.00%	25.00%	66.70%	8.30%	
22yrs	0.00%	14.30%	42.90%	42.90%	0.00%	

**Table 4:** Gender and Grittiness

**GENDER**	**Level of Grittiness**					
	**Not at all Gritty**	**Somewhat Gritty**	**Gritty**	**Very much Gritty**	**Extremely Gritty**	**P value**
Female	0	1%	35.00%	57.50%	5.00%	0.961
Male	0	15.80%	47.40%	26.30%	10.50%	
